# Orthographic Transparency Enhances Morphological Segmentation in Children Reading Hebrew Words

**DOI:** 10.3389/fpsyg.2017.02369

**Published:** 2018-01-19

**Authors:** Laurice Haddad, Yael Weiss, Tami Katzir, Tali Bitan

**Affiliations:** ^1^Department of Communication Sciences and Disorders, University of Haifa, Haifa, Israel; ^2^Department of Psychology, University of Texas at Austin, Austin, TX, United States; ^3^Department of Learning Disabilities and The Edmond J. Safra Brain Research Center for the Study of Learning Disabilities, University of Haifa, Haifa, Israel; ^4^Department of Psychology, IIPDM, IBBR, University of Haifa, Haifa, Israel; ^5^Department of Speech-Language Pathology, University of Toronto, Toronto, ON, Canada

**Keywords:** morphological segmentation, reading acquisition, orthographic transparency, Hebrew, root, diacritic marks, children, reading

## Abstract

Morphological processing of derived words develops simultaneously with reading acquisition. However, the reader’s engagement in morphological segmentation may depend on the language morphological richness and orthographic transparency, and the readers’ reading skills. The current study tested the common idea that morphological segmentation is enhanced in non-transparent orthographies to compensate for the absence of phonological information. Hebrew’s rich morphology and the dual version of the Hebrew script (with and without diacritic marks) provides an opportunity to study the interaction of orthographic transparency and morphological segmentation on the development of reading skills in a within-language design. Hebrew speaking 2nd (*N* = 27) and 5th (*N* = 29) grade children read aloud 96 noun words. Half of the words were simple mono-morphemic words and half were bi-morphemic derivations composed of a productive root and a morphemic pattern. In each list half of the words were presented in the transparent version of the script (with diacritic marks), and half in the non-transparent version (without diacritic marks). Our results show that in both groups, derived bi-morphemic words were identified more accurately than mono-morphemic words, but only for the transparent, pointed, script. For the un-pointed script the reverse was found, namely, that bi-morphemic words were read *less* accurately than mono-morphemic words, especially in second grade. Second grade children also read mono-morphemic words faster than bi-morphemic words. Finally, correlations with a standardized measure of morphological awareness were found only for second grade children, and only in bi-morphemic words. These results, showing greater morphological effects in second grade compared to fifth grade children suggest that for children raised in a language with a rich morphology, common and easily segmented morphemic units may be more beneficial for younger compared to older readers. Moreover, in contrast to the common hypothesis, our results show that morphemic segmentation does not compensate for the missing phonological information in a non-transparent orthography, but rather that morphological segmentation is most beneficial in the highly transparent script. These results are consistent with the idea that morphological and phonological segmentation processes occur simultaneously and do not constitute alternative pathways to visual word recognition.

## Introduction

Work in recent years demonstrates the critical contribution of morphological processing abilities to reading and reading acquisition (Treiman and Cassar, 1996; McBride-Chang et al., 2003; Deacon and Kirby, 2004; Kieffer and Lesaux, 2012). Morphological processing ability is the reader’s sensitivity to the smallest units of meaning in words, the ability to extract roots and affixes from whole-words and manipulate them to produce grammatically correct words. When these manipulations are performed intentionally, or through conscious reflection, they are referred to as ‘morphological awareness’ (Carlisle and Nomanbhoy, 1993; Kuo and Anderson, 2006; Bowers et al., 2010; Carlisle, 2010; Nagy et al., 2014).

While there is a growing body of literature on the strong relationships between morphological skills and reading, it is not yet clear how morphological segmentation processes interact with phonological processing during reading and reading development (Carlisle and Nomanbhoy, 1993; Fowler and Liberman, 1995). The effect of morphological skills on reading changes with age and reading experience (Casalis and Louis-Alexandre, 2000; Carlisle and Stone, 2005; Marcolini et al., 2011). Moreover, the degree to which morphological segmentation processes facilitates visual word recognition during reading depends on the morphological properties of the language (Marslen-Wilson et al., 1994; Bertram et al., 1999; Rispens et al., 2008; Duncan et al., 2009; Tolchinsky et al., 2012), on morphological transparency of the orthography (the degree to which derived words perserve the form of the morphemic units; Clahsen et al., 1997; Carlisle and Stone, 2005), and on the phonological consistency of the orthography (Frost, 2012; Casalis et al., 2015). A language with a rich morphology may promote strong reliance on morphological processes already at early stages of reading acquisition. One common hypothesis is that morphological decomposition is especially helpful in deep orthographies, where there is no consistent mapping of graphemes to phonemes, because it compensates for the scarce phonological information (Bar-On and Ravid, 2011; Vaknin-Nusbaum and Miller, 2011). In contrast, in a shallow orthography, where readers can rely on the direct correspondence of letters to sounds, the reliance on morphemes to access meaning is expected to be low (Frost, 2006).

Hebrew has two interesting properties: a rich Semitic morphological system, in which most words are composed of a root and a morphemic pattern, and a dual version of orthography one transparent and one opaque. These characteristics provide an opportunity to examine the interaction between orthographic transparency and morphological complexity on reading processes among developing readers in a within language design. The goal of the current study is to examine the effect of the phonological information present in the script on children’ tendency to engage on morphological segmentation in Hebrew speakers. Specifically, we aim to determine (a) The contribution of basic morphological derivation processes to word reading rate and accuracy in Hebrew speaking second and fifth grade children; (b) Whether this contribution differs between transparent and non-transparent scripts; and (c) whether the involvement of morphological processes in reading changes with reading experience and age.

### Engagement of Morphological Processing in Word Recognition in Children

As the smallest meaning-bearing linguistic unit, morphemes have the potential to serve as the elementary building blocks of word representations, supporting an economical, non-redundant body of lexical knowledge that facilitates the learning of novel forms and morphological variants of known words (e.g., Rastle and Davis, 2008; Merkx et al., 2011). However, while the role of *phonological* awareness in reading acquisition has been extensively studied for three decades and shown in a variety of orthographies (e.g., Vellutino and Scanlon, 1987; Ben-Dror et al., 1995; Ziegler et al., 2010; Melby-Lervag et al., 2012), the importance of *morphological* segmentation skills to reading development was the focus of researchers’ attention mainly in the last decade (Carlisle and Nomanbhoy, 1993; Carlisle, 2000; Deacon and Kirby, 2004). The notion is that children’s ability to recognize familiar morphemes embedded in morphologically derived and inflected words facilitates their ability to recognize written words. Although some of the earlier studies (Fowler and Liberman, 1995; Shankweiler et al., 1995) suggested that the role of morphological awareness in reading is attributed to its covariance with phonological awareness, more recent studies showed the unique contribution of morphological awareness to reading achievements in children (Casalis and Louis-Alexandre, 2000; Mahony et al., 2000; Nagy et al., 2003; Deacon and Kirby, 2004).

While there is no question today about the importance of morphological knowledge for reading acquisition, the developmental trajectory of morphological knowledge and its effect on reading acquisition is less clear. Morphological knowledge of spoken language develops over time, through accumulating language experience. Children, as young as 3 years of age are aware of the morphological components of words and how they can be manipulated to create new words (Berman, 1982). Studies in English speaking children show that morphological awareness of inflections and simple derivations might emerge early whereas an understanding of more complex derivational relations may come into place later. For example, while kindergarten and first grade children display some competence with simple derivations that do not involve phonological changes in the morphemes (Clark and Cohen, 1984), older children adeptly tackle more complex derivational relations (such as between electric and electricity), by about the fourth grade (Carlisle, 1988; Tyler and Nagy, 1989). As in spoken language, children’s ability to extract morphemes from written words increases simultaneously with the improvement in reading accuracy, fluency and comprehension during elementary school years (Kieffer and Lesaux, 2012; Nagy et al., 2014; Sparks and Deacon, 2015). Children’s performance on morphological awareness tasks (e.g., judgment of decomposability and defining the correct usage of complex words) increases from first to third grade (Carlisle and Fleming, 2003). Share (1995) suggests that recognition of morphemic regularities is an indication of a child’s consolidation and fluency of print-sound correspondences.

Many studies suggest that in addition to the improvement of morphological awareness and segmentation skills, there is also an increase in the contribution of these abilities to reading in later stages of reading acquisition, mainly due to the increase in the proportion of complex words in the lexicon (Adams, 1990; Anglin, 1993; Mahony et al., 2000; Singson et al., 2000; Kuo and Anderson, 2006; Rispens et al., 2008). A study of French speaking children (Casalis and Louis-Alexandre, 2000) showed that morphological awareness had a significant contribution to the variance in words decoding skills, in second grade but not in first grade. However, readers’ reliance on morphological segmentation does not depend only on their morphological knowledge, but also on the complete set of reading skills available to them while trying to identify written words. Hence, morphological segmentation can serve as a compensatory reading strategy for children and adults with low reading skills, who do not fully master whole-word processing, with a decrease in reliance on morphology in more skilled readers. For example, Italian speaking children and adult with dyslexia benefit from the morphological structure of derived words in an oral reading task more than skilled adult readers (Burani et al., 2008; Marcolini et al., 2011). Similarly, a study in French showed that the morphemic status of words had a facilitative effect on spelling only in poor readers but not in skilled readers (Quemart and Casalis, 2017). Other studies, in English and French (Nunes et al., 2003; Carlisle and Stone, 2005; Quemart et al., 2011), show the contribution of morphological knowledge to reading acquisition already at the beginning of elementary school and suggest that some morphological regularities may have very early effects on reading, and some may even have greater effects in early compared to later stages of reading development.

In addition to the individual’s reading skills, the reliance on morphemic units during reading also depends on the morphological structures of the language (Marslen-Wilson et al., 1994; Bertram et al., 1999), as well as the transparency of orthography to phonology correspondence (Clahsen et al., 1997; Carlisle and Stone, 2005; Frost, 2012; Casalis et al., 2015). A study that tested the effect of morphological cues on spelling in school-age children (1–6 grade) found a greater reliance on morphological cues in Hebrew compared to Dutch speaking children (Gillis and Ravid, 2006), presumably due to the rich Semitic morphology of the Hebrew language. It has been suggested that morphological decomposition may compensate for incomplete phonological information in opaque orthographies in which the phonological code cannot be easily accessed through mapping of smaller units (Ziegler et al., 1997; Frost, 2006; Bar-On and Ravid, 2011; Vaknin-Nusbaum and Miller, 2011). However, a study that compared French and English speaking children showed stronger morphological effects in French, which has a more transparent orthography (Casalis et al., 2015). The authors suggest that the rich morphological productivity in French has outweighed the effect of the opaque English orthography. The unique properties of the Hebrew orthography provide an opportunity to test the complex interactions between morphological complexity and orthographic transparency in a within language and within subject design.

### Hebrew Orthography and Morphology

Hebrew has one script with two versions that differ in their orthographic transparency. The opaque version is the un-pointed “Abjad” orthography that represents mostly consonants, and partially represents vowels using vowel letters. Vowel letters, provide only ambiguous vowel information because they denote both consonants and vowels, and some of them represent more than one vowel, creating extensive phonological under-specification (Bar-On, 2010). The transparent version is pointed, with diacritic marks superimposed under or above the letters, providing full representation of words’ phonology. Children learn to read the transparent (pointed) version first, and are only exposed to the un-pointed version around 2nd or 3rd grade, with the transformation to the un-pointed script completed around 4th grade (Bar-On and Ravid, 2011).

The Hebrew language is also characterized by high morphological density in both its inflectional and derivational word formation. In the Hebrew derivational system, as in other Semitic languages, most words are morphologically complex as they are composed of two abstract morphemes: the root and the word pattern (Mishkal/Binyan). All the verbs and most of the nouns and adjectives in Hebrew are derived via non-linear formation in which a consonantal root is interleaved with a vocal pattern which adds the vowels between the root consonants [i.e., “GIDUL”, “
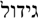
”, (*growth*) root – G.D.L. (*associated with growing*) pattern CiCuC] (Ravid and Malenky, 2001). The root that provides the basic meaning to the word, is not an independent word. It typically consist of three (but sometimes four) consonants. The morphemic pattern consists of the vowels, and can also include consonants, but only at the beginning [e.g., “MIGDAL”, “
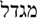
”, (*tower*) root – G.D.L. pattern miCCaC] and/or at the end [e.g., “MAGDELET”, “
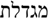
”, (*magnifying (glass)*) root – G.D.L. pattern maCCeCet] of the word. Thus, the orthographic representation of the root is an almost continuous sequence, which is only interrupted occasionally by narrow vowel letters (vav- “
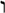
”, which represents the vowels /o/ and /u/, or yod-“
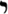
” which represent the vowel /i/). Other vowels (i.e., /a/ or /e/ which can be represented by the letters he’-“
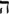
” or aleph-“
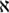
”) are never represented by a vowel letter when they appear amidst the root consonants (Ravid and Malenky, 2001; Vaknin-Nusbaum et al., 2015).

### The Effect of Diacritics and Derivational Morphology on Reading in Hebrew

Diacritics facilitate word recognition in early stages of reading acquisition (Navon and Shimron, 1981; Shimron and Sivan, 1994; Ravid, 1996; Harel-Koren, 2007; Shany and Share, 2011). In skilled adult readers diacritics have mixed effects, showing either facilitation (Navon and Shimron, 1981; Shimron and Navon, 1982; Koriat, 1984, 1985) or no effect (Bentin and Frost, 1987; Shimron and Sivan, 1994; Schiff and Ravid, 2004; Harel-Koren, 2007) on speed and accuracy of word recognition. However, both behavioral (Weiss et al., 2015a) and brain imaging (Weiss et al., 2015b) studies, of word reading in Hebrew show that even skilled adult readers who do not benefit from diacritics in terms of accuracy or RT, resort to a more piecemeal segmentation approach of decoding small units when reading pointed words.

Morphological segmentation has a prominent role in reading Hebrew words. Studies in skilled adult readers found long lasting priming effects of both the root and the morphemic pattern, suggesting that these units are activated when skilled Hebrew readers recognize written words (Bentin and Feldman, 1990; Frost et al., 1997). Studies in Hebrew speaking children show that children’s awareness of the root morpheme in spoken language is already evident at the age of 3 years (Berman, 1982), however, the awareness of patterns develops later, and only reaches an adult level in 9th grade (Ravid and Malenky, 2001). School children’s (2nd–6th grades) performance in morphological analogies of *written* words and pseudowords also shows early robust explicit knowledge of roots and patterns (Ravid and Schiff, 2006). Furthermore, children’s morphological awareness in explicit judgment tasks was shown to correlate with their reading skills in 2nd (Vaknin-Nusbaum et al., 2015, 2016a,b) and 5th grade (Cohen-Mimran, 2009; Vaknin-Nusbaum et al., 2015), suggesting that morphological knowledge affects children’s ability to read Hebrew already in early stages of reading acquisition. The triplex model of Hebrew reading development (Share and Bar-On, 2017) denotes three phases of development with growing sizes of units: the phonological (*sub-lexical*) sequential spelling-to-sound translation (in Grade 1); the higher-order string-level (*lexical*) lexico-morpho-orthographic processing (in Grade 2) followed by a *supra-lexical* contextual level (in upper elementary grades). According to this model first grade readers practice their decoding skills in reading phonologically transparent script. In second grade, readers gradually begin using their lexico-morpho-orthographic knowledge to cope with the incompletely vocalized Hebrew, a process which promotes their reading fluency. Then, during third grade and the transition to the unpointed script readers must rely on context in order to solve ambiguities, due to the extensive homography (Share and Bar-On, 2017).

While many of the above studies used explicit judgment tasks of morphological awareness, another study examined morphological segmentation skills in an online reading task of unfamiliar words and pseudowords. Hebrew speaking children (2nd to 11th grade) read aloud unfamiliar words and pseudowords containing real morphemic patterns, written in the opaque un-pointed script (Bar-On and Ravid, 2011). The results show a dramatic improvement in children ability to rely on morphosyntactic cues to identify unfamiliar words during 2nd grade, with a more gradual improvement throughout the rest of the school years. This study shows that Hebrew speaking children are able to extract morphological patterns from written words in very early stages of reading acquisition and this knowledge facilitates identification of new words. However, it is not clear to what extent these segmentation skills are also applied when reading real and familiar words. Moreover, the authors note that this ability facilitates reading of the opaque un-pointed script. However, since this study did not compare pointed and un-pointed words, it is not clear whether the reliance on morphological segmentation was indeed enhanced by the opacity of the un-pointed script.

### The Current Study

The goal of the current study is to examine the interaction between morphological and phonological segmentation processes during reading development. For this aim we tested the effect of orthographic transparency on children’s reliance on morphological segmentation when reading Hebrew derived words. Children (in 2nd and 5th grade) read single words (nouns) presented with or without diacritic marks, and the reliance on morphological segmentation was tested by comparing the speed and accuracy of reading bi-morphemic words (composed of a productive root and pattern) and mono-morphemic words. These two age groups represent lower and higher reading skills and reading experience within elementary school, and specifically 2nd grade children have a very a low level of experience with the unpointed script.

Many of the previous studies that tested the effect of morphological knowledge on reading development in Hebrew (Cohen-Mimran, 2009; Vaknin-Nusbaum et al., 2015) and other languages (Carlisle, 2000; Deacon and Kirby, 2004; Kieffer and Lesaux, 2012) used an explicit measure of morphological awareness. By comparing between oral reading of bi-morphemic and mono-morphemic words the current study tests the reliance on morphological segmentation in an online method. This approach, which was previously used in studies of Italian speakers (Burani et al., 2008; Marcolini et al., 2011) measures the reader’s sensitivity to the words morphological structure implicitly and directly, without relying on their meta-linguistic awareness. This approach may be better suited to tapping young children’s mental processes.

Based on previous studies, showing the sensitivity of young Hebrew speakers to roots and patterns in spoken and written derived words (Berman, 1982; Bar-On and Ravid, 2011) we expect that: (1) the morphological structure will facilitate word recognition as would be evident in higher accuracy of bi-morphemic compared to mono-morphemic word reading. (2) This effect is expected to be stronger in 2nd grade compared to 5th grade children, because the older children may rely more on whole-word recognition when reading familiar words (Burani et al., 2008; Marcolini et al., 2011). (3) Finally, based on the notion that reliance on morphological segmentation can compensate for the sparse phonological information in opaque orthographies (Ziegler et al., 1997; Frost, 2006; Bar-On and Ravid, 2011), we predict that the facilitating effect of the morphemic structure would be stronger in un-pointed compared to pointed words.

## Materials and Methods

### Participants

Twenty-nine 2nd grade students (ages 7:01–8:04 years, 16 girls) and 29 5th grade students (ages 10:01–11:04 years, 17 girls) were recruited from a regular elementary school in north Israel. Written informed consent was obtained from the parents of all participants. The study was approved by the ethics committee of the Faculty of Social Welfare and Health Sciences at the University of Haifa, and by the Ministry of Education. All were native Hebrew speakers without learning disabilities as reported by their teachers and confirmed by our assessment. Their reading level was tested using the Word Recognition and the Pseudo Word Decoding Tests, from “*Alef-Taf, Diagnostic test battery for written language disorders*” (Shany et al., 2006) described below. The inclusion criterion was having a score higher than one standard deviation below the norm in both tests and both measures: reading rate and accuracy. No student was excluded based on this criterion. One 2nd grade participant was excluded from the group analysis because their performance on the experimental task was lower than 3 standard deviations below the group average in three of the four experimental conditions, in both accuracy and reaction time. This resulted in 28 participants in 2nd grade and 29 participants in 5th grade who were included in the analysis.

### Stimuli

The experimental stimuli consist of 96 concrete Hebrew nouns in two levels of orthographic transparency and two morphological conditions. Morphologically rich (bi-morphemic) words are composed of two morphemes: a root + a morphemic pattern. Examples of the stimuli are shown in **Table 1**. All roots were three consonantal productive roots, which are also used in existing Hebrew verbs, as judged by a linguist. Morphologically simple (mono-morphemic) words cannot be decomposed into smaller morphemes. We did not include words that can be decomposed into base + suffix (e.g., /gagon/: /gag/+/on/ ‘small roof’) even if they did not include a root.

**Table 1:** Example of stimuli.

	Bi-morphemic (root and template)	Mono-morphemic
With Diacritics	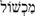	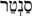
	MXSOL	SNTR
	/mixshol/	/santer/
	(obstacle)	(chin)
Without diacritics	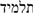	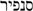
	TLMID	SNPIR
	/talmid/	/snapir/
	(student)	(fin)


In each morphological level, half of the words (24) were presented with diacritics and half without diacritics. None of the words in the experiment were homographs even when presented with no diacritics. Word lists were matched across conditions for the number of consonants (3–4), the number of vowel letters (0–2), the number of syllables (2–3), and for written frequency. As there is no available consensus corpus for written Hebrew frequency, we based the frequency ranking on the rating of ten elementary school teachers on a Likert scale of 1–5, that represents a range of low to high frequency for second graders. The frequency of the selected words ranged from 2 to 4.8, and the average frequency was equal in all conditions (between 3.4 and 3.6).

### Standardized Tests

All participants underwent three standardized screening tests, in order to assess their reading and decoding abilities, and their vocabulary knowledge. In addition, phonological and morphological abilities were measured in order to assess the correlation of these abilities with performance on the experimental task. The first two screening tests were taken from the “*Alef-Taf*” battery (Shany et al., 2006): (1) *Word recognition*: participants read aloud 38 nouns with diacritics which represent different levels of frequency, length, and phonological structure. Different age-appropriate lists are used for different age groups. The scores indicate the number of accurately read words per minute and the percentage of errors. (2) *Pseudo-word decoding:* participants read aloud 33 pseudo-words with diacritics. 24 of these items represent familiar morpho-phonological structures in Hebrew and nine contained sound structures non-existent in Hebrew. Different age-appropriate lists are used for different age groups. The obtained scores indicate the number of accurately read pseudowords per minute and the percentage of errors. (3) *Vocabulary subtest* (WISC-III) (Wechsler, 1994): participants provided the definition for 25 words presented orally by the examiner. The final score is the total number of correctly defined words.

One phonological awareness and two morphological awareness tests were included in order to test the correlation between performance on the experimental task and these abilities: (1) *Phonological awareness* (from the “Alef-Taf” battery; Shany et al., 2006): includes 16 mono and bi-syllable words read aloud by the examiner. Participants produce pseudo-words obtained by omitting a designated phoneme positioned at the beginning, middle or end of the word. The score reflects the percentage of correctly produced items. (2) *Morpho-syntactic awareness* (from the “Alef-Taf” battery; Shany et al., 2006)*:* the ability to inflect and derive words in a context of a sentence is tested in both identification and production. *Identification*–participants identify the missing word in a sentence from five given options, which are all morphologically related to the target word. The score indicates the error percentage in the task. *Production* –participant s are required to complete a missing word in a sentence by deriving it from a given root. Other items include completing a missing pseudo-word in a sentence by inflecting a given pseudo-root. (3) *Passive production test* (Ravid and Saban, 2008). The examiner reads aloud a sentence and the participant is required to say it in the passive form. The test includes 12 sentences and the score represents the percentage of correct items.

### Experimental Procedure

We employed an oral naming task because of its high ecological validity for testing reliance on morphological and phonological representations during word recognition (Koriat, 1984, 1985; Burani et al., 2008). Stimuli were presented on a computer monitor and participants were required to read them aloud, while responses and reaction times were recorded using a voice-activated-key (E-prime, Serial Response Box, PST). The trial began with the presentation of a fixation cross, and the presentation of the word was triggered by the participant. The word appeared on the screen 250 ms after the button press and remained there until 1200 ms after the onset of the vocal response when it was replaced by a fixation cross. Reaction times (RT) were collected starting from the stimulus presentation to the onset of vocalization. The 96 words from the current experiment were presented together with 152 words from another experiment (see Weiss et al., 2015a,b) which were similar in length and frequency and also appeared in both the pointed and un-pointed versions. Hence, the total number of trials for both experiments together was 248. Pointed and un-pointed words were presented in separate blocks of 124 words each, to minimize interference from frequent switching between strategies associated with reading pointed and un-pointed words. Block order was counter balanced across individuals, while morphological complexities were randomly intermixed. Data were collected in two sessions during the second trimester of the school year. In the first session the participants performed the standardized tests individually in a quiet room in the school. Participants who passed the selection criteria were invited for a second session where they performed the experimental task.

### Data Analysis

Reaction time was analyzed only for correct responses. Self-corrected responses and words read by sounding each letter separately were coded as correct for the analysis of accuracy but were omitted from the analysis of reaction time. 1% of the responses were excluded from the analysis of RT due to technical recording problems. In order to examine the effects of diacritics and morphological complexity on reading, statistical analysis incorporated a GLM analysis separately on response time and accuracy as dependent variables with morphological complexity (mono-morphemic vs. bi-morphemic) and diacritics (pointed vs. un-pointed) as within subject variables and grade (2nd vs. 5th) as between subject variable. In order to test our specific hypotheses within each age group, we have also conducted separate analyses within each group although the interaction with group was not significant. Finally, in order to examine the linguistic and cognitive bases for the effects of diacritics and morphological complexity found in our experimental manipulation we have included scores of three standardized measure (Passive production test, Morpho-syntactic awareness and Phonological awareness) as covariates in three separate GLM analyses, conducted as above across both groups. Significant effects are reported with *p* < 0.05.

## Results

### Characteristics of the Sample

Performance of all participants was within one standard deviation from the mean in all screening measures (accuracy and speed of words recognition and pseudo-words decoding, and vocabulary) as computed based on the age-appropriate norms of these standardized tests. These scores are presented in **Table 2**. Raw scores are presented in Supplementary Table S1. The scores of the other tests which were used for the correlation analysis are presented in **Table 3**.

**Table 2:** Participants’ average *Z* score (and SD) in the screening tests.

	2nd grade (*n* = 28)	5th grade (*n* = 29)
Reading pseudo-words	1.01 (0.55)	There are no norms
(% errors *z* score)		
Reading pseudo-words	0.79 (0.56)	0.96 (0.56)
(number per minute z score)		
Reading words	0.89 (0.33)	0.56 (0.39)
(% errors *z* score)		
Reading words	0.98 (0.67)	0.17 (0.72)
(number per minute *z* score)		
Vocabulary	13.18 (2.80)	12.21 (2.02)
(scaled score)		


*For the vocabulary test the scaled scores are presented (mean = 10, *SD* = 3).*

**Table 3:** Participants’ average scores (and SD) in the Phonological and Morphological awareness tests used in the correlation analysis.

	2^nd^ grade (*n* = 28)	5^th^ grade (*n* = 29)
Phoneme omission	22.10 (26.92)	9.70 (14.99)
(% errors)	47.49 (26.89)	24.50 (24.35)
Morpho-syntactic awareness	12.69 (10.78)	6.51 (5.95)
(% errors)	There are no norms	10.51 (11.12)
Passive production	65.47 (20.38)	76.72 (12.87)
(% accuracy)	61.04	89.37


*The mean and standard deviation of the norm sample appear below.*

### Experimental Task

#### Accuracy

**FIGURE 1:**
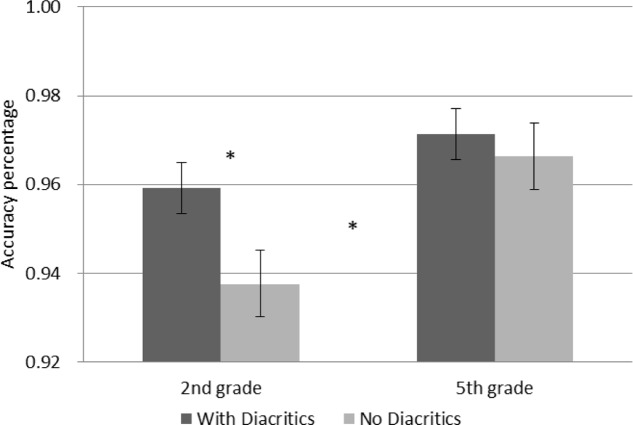
Accuracy in reading words with and without diacritics according to grade. Error bars indicate standard errors. Asterisks represent significant differences at *p* < 0.05.

**FIGURE 2:**
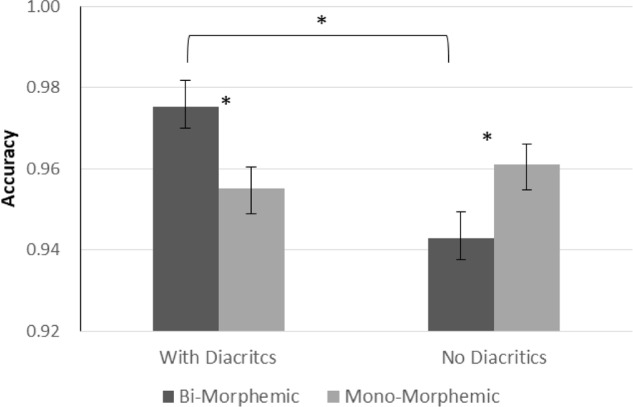
Interaction between Morphological complexity and Diacritics across groups. Error bars indicate standard errors. Asterisks represent significant differences at *p* < 0.05.

A GLM repeated measures analysis for accuracy as the dependent variable was conducted with morphological complexity (mono-morphemic vs. bi-morphemic words) and diacritics (with/without diacritics) as within-subject variables and grade (2nd vs. 5th) as a between subject factor. The analysis showed significantly more accurate performance in 5th grade [*F*(1,54) = 6.538, *p* = 0.013], and a significant effect of diacritics [*F*(1,54) = 6.697, *p* = 0.012] showing that diacritics improved accuracy across groups (see **Figure 1**). The analysis also showed a significant interaction between diacritics and morphological complexity [*F*(1,54) = 14.919, *p* < 0.001], and no interactions with grade. **Figure 2** present follow-up analyses across the two groups showing that for the pointed script bi-morphemic words were read significantly more accurately than mono-morphemic words [*t*(55) = 3.154, *p* = 0.003], while for the un-pointed script the pattern was reversed, with bi-morphemic words read *less* accurately than mono-morphemic words [*t*(55) = (-2.569), *p* = 0.013]. The results also showed that the pointed script improved accuracy compared to un-pointed script only for bi-morphemic words [*t*(55) = 4.139, *p* < 0.001], with no effect on mono-morphemic words [*t*(55) < 1, NS].

**FIGURE 3:**
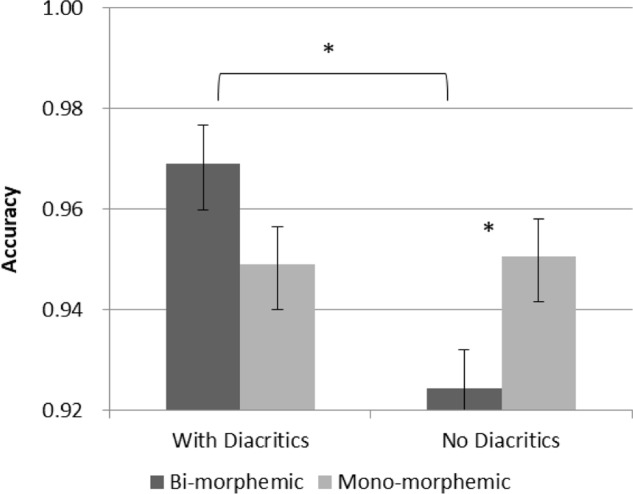
Accuracy in 2nd grade. Error bars indicate standard errors. Asterisks represent significant differences at *p* < 0.05.

**FIGURE 4:**
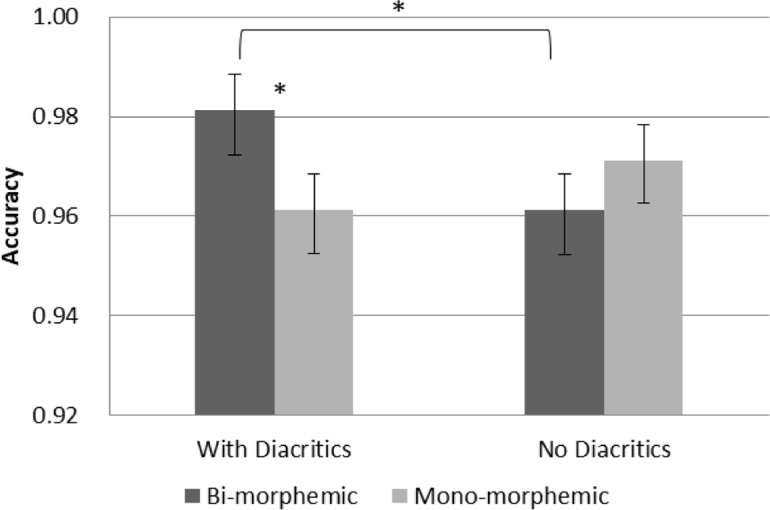
Accuracy in 5th grade. Error bars indicate standard errors. Asterisks represent significant differences at < 0.05.

Although no interaction with group was significant, in order to see whether the above effects are evident in both age group and test our specific hypotheses, separate analyses were conducted within each age groups (see **Figures 3**, **4**). The results show that the main effect of diacritics was only significant in 2nd grade [*F*(1,26) = 6.041, *p* = 0.021], however, the interaction between diacritics and morphological complexity was significant in both groups [2nd grade: *F*(1,26) = 7.692, *p* = 0.01; 5th grade: *F*(1,28) = 7.368, *p* = 0.011], with a similar pattern of results across groups (see **Figures 3**, **4**). For the pointed script bi-morphemic words were read more accurately than mono-morphemic words [significant in 5th grade (*t*(28) = 2.544, *p* = 0.017) and marginally significant in 2nd grade (*t*(26) = 1.951, p = 0.062)]. Only 2nd graders showed the opposite pattern for the un-pointed script, with lower accuracy for bi-morphemic compared to mono-morphemic words [*t*(26) = -2.185, *p* = 0.038]. The two groups also showed a similar pattern of effects within each level of morphological complexity: the pointed script improved reading accuracy compared to the un-pointed script only for bi-morphemic words [2nd grade: *t*(26) = 3.357, *p* = 0.002; 5th grade *t*(28) = 2.544, *p* = 0.017], but had no effect on mono-morphemic words in any age group.

#### Reaction Time

**FIGURE 5:**
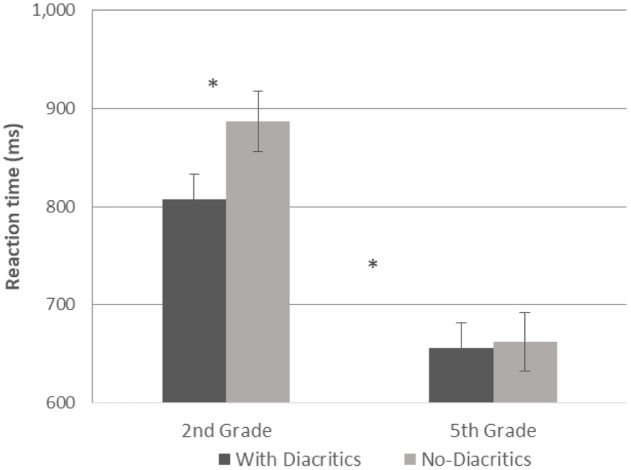
Reaction time in pointed and un-pointed words for both groups.

**FIGURE 6:**
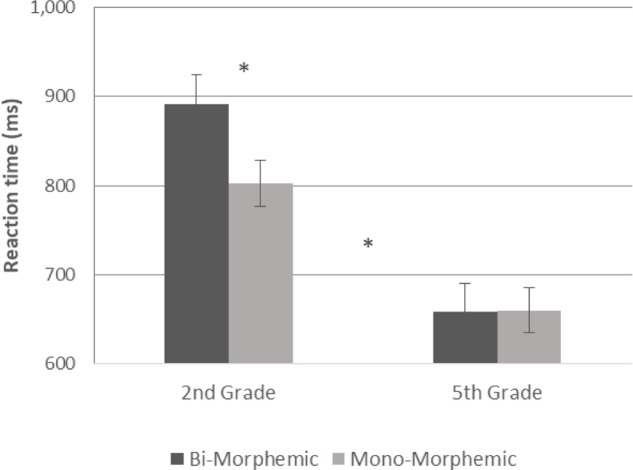
Reaction time in mono-morphemic and bi-morphemic words in both groups.

**FIGURE 7:**
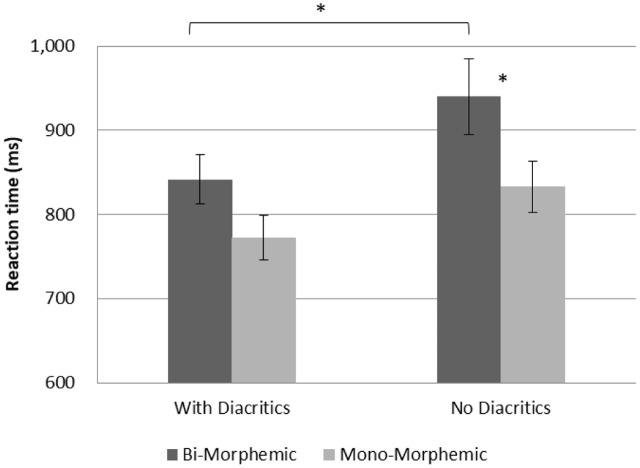
Reaction time in 2nd grade.

A GLM repeated measures analysis was conducted with reaction time as the dependent variable, morphological complexity and diacritics as within subject variables and with grade as between subject factor. The analysis showed a significant effect for grade [*F*(1,54) = 23.394, *p* < 0.001], with faster responses in 5th grade (see **Figures 5**, **6**). It also showed a significant effect for diacritics [*F*(1,54) = 4.963, *p* = 0.03], with faster responses for the pointed script, and a significant effect for morphological complexity [*F*(1,54) = 8.785, *p* = 0.005] with faster responses for mono-morphemic words. The two-way interaction for grade and morphological complexity was also significant [*F*(1,54) = 9.712, *p* = 0.003] and the interaction between grade and diacritics was marginally significant [*F*(1,54) = 3.693, *p* = 0.06]. These interactions were followed-up by analyses within each grade level, showing that both of these effects were only significant in the 2nd grade [morphological complexity: *F*(1,26) = 8.968, *p* = 0.006; Diacritics: *F*(1,26) = 4.440, *p* = 0.045]. Hence, for 2nd grade children reaction time was faster for pointed compared to un-pointed words (**Figures 5**, **7**) and for mono-morphemic compared to bi-morphemic words (**Figures 6**, **7**), with no effects in 5th grade.

### Correlations with Standardized Tests

#### Passive Production Test (Ravid and Saban, 2008)

In order to determine the linguistic mechanism underlying the effects in our experimental task, three linguistic measures were included in the GLM repeated measures analyses of accuracy as covariates. The inclusion of the Passive production test as a covariate in the GLM analysis of accuracy across groups showed a main effect for the Passive production test [*F*(1,53) = 15.985, *p* < 0.001], and no interaction with other factors. This was due to positive correlations between performance on the Passive production test and accuracy on the experimental task, which was significant in three of the four conditions (unpointed bi-morphemic words: *r* = 0.410, *p* = 0.002; un-pointed mono-morphemic words *r* = 0.442, *p* = 0.001; pointed bi-morphemic words: *r* = 0.399, *p* = 0.002). None of the other effects were significant (including those which were significant in the original analysis, namely, diacritics, group and morphology × diacritics). These findings indicate that participants with high morphological awareness as measured by the Passive production test, generally read words more accurately.

#### Morpho-Syntactic Awareness (Shany et al., 2006)

**FIGURE 8:**
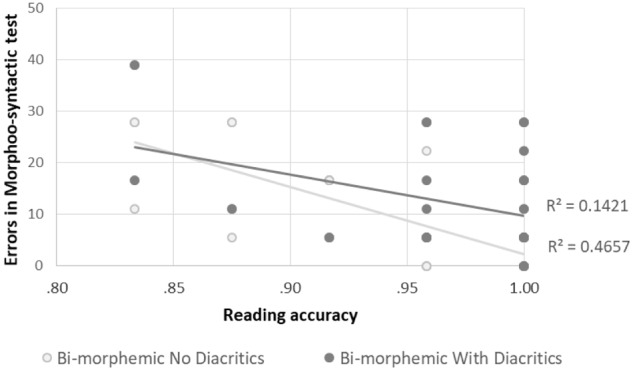
The correlation between scores in the Morpho-syntactic awareness test and reading accuracy in bi-morphemic words in 2nd grade children.

The inclusion of the Morpho-syntactic awareness test scores as a covariate in the GLM analysis of accuracy across groups showed a main effect for the Morpho-syntactic awareness test [*F*(1,53) = 5.907, *p* = 0.019]. There were also significant interactions between the score of the Morpho-syntactic awareness test and the experimental manipulation of morphological complexity [*F*(1,53) = 4.231, *p* = 0.045], as well as between the Morpho-syntactic awareness test and grade [*F*(1,53) = 4.298, *p* = 0.043], indicating that the effect of the morpho-syntactic awareness test is different for the two grade levels. In addition, only the interaction of morphology with diacritics remained significant as in the original analysis [*F*(1,53) = 7.735, *p* = 0.007], while the other effects (diacritics and group) were not. Separate GLM analyses were therefore conducted in each grade level. Only 2nd grade children showed a significant effect of the Morpho-syntactic awareness test [*F*(1,25) = 12.229, *p* = 0.002], and an interaction between the morpho-syntactic awareness test and the experimental task morphological complexity [*F*(1,25) = 6.347, *p* = 0.019]. No effects involving the Morpho-syntactic test were found among 5th graders [*F*(1,27) = 0.421, NS]. Pearson correlation analyses within the 2nd grade group showed negative correlations between the morpho-syntactic test scores (expressed as error percentage) and reading accuracy in bi-morphemic un-pointed words (*r* = -0.682, *p* < 0.001) and marginally significant in bi-morphemic pointed words (*r* = -0.377, *p* = 0.053). The correlations with mono-morphemic words were not significant (*r* = -0.179, *p* = 0.372; *r* = -0.269, *p* = 0.175; for the pointed and un-pointed scripts respectively). Namely, the Morpho-syntactic awareness test predicted reading accuracy only for 2nd grade children and only for bi-morphemic words (see **Figure 8**).

### Phonological Awareness

Including the Phoneme omission test scores as a covariate in the GLM analysis of accuracy or RT across groups showed no significant effect of the test [*F*(1,53) = 1.068, 1.125, NS for accuracy and RT respectively].

## Discussion

Our study examined the hypothesis that the morphological structure of root and pattern, in derived Hebrew words, would facilitate children’s oral reading, as compared to mono-morphemic words. We further expected that this facilitation would be stronger in 2nd grade compared to 5th grade children, and stronger in the opaque, un-pointed compared to the transparent pointed script.

Our results showed faster and more accurate reading in 5th grade compared to 2nd grade children. The transparent, pointed script, was read faster and more accurately compared to the un-pointed script, in 2nd grade children. 2nd grade children also read mono-morphemic words faster than bi-morphemic words. Interestingly we found an interaction between morphology and diacritics across both groups. Specifically, in both groups, derived bi-morphemic words were identified more accurately than mono-morphemic words, but this was only true for the transparent, pointed, script. For the un-pointed script the reverse was found, namely that bi-morphemic words were read *less* accurately than mono-morphemic words, especially in 2nd grade.

Correlation analyses with morphological awareness tests showed a correlation between reading accuracy across conditions and performance on the Passive production test (Ravid and Saban, 2008) across both groups. Interestingly, the Morpho-syntactic awareness test from the Alef-Taf battery (Shany et al., 2006) was specifically associated with reading accuracy of bi-morphemic words and only among 2nd grade children. The following sections will discuss these results and their implications for our understanding of morphological segmentation during reading development in transparent and opaque scripts.

### Morphological Segmentation and Its Interaction with Orthographic Transparency

The finding that across both groups, bi-morphemic words were read more accurately than mono-morphemic words, in the pointed script, is partly consistent with our hypothesis. We predicted that the morphological structure of root and pattern would facilitate word identification in general, but more so in unpointed words. The facilitation found in pointed words is consistent with findings in Italian, a transparent orthography, showing higher accuracy in oral reading of bi-morphemic compared to mono-morphemic words (Burani et al., 2008; Marcolini et al., 2011). The authors suggest that morphological segmentation facilitates reading by providing smaller units, which could be identified with a shorter visual span. However, in contrast to our hypothesis that morphological decomposition would be more beneficial for *unpointed* words, because it could compensate for the scarce phonological information (Ziegler et al., 1997; Frost, 2006; Bar-On and Ravid, 2011), our results revealed the opposite pattern. Bi-morphemic words were read *less* accurately than mono-morphemic words in the un-pointed script for 2nd grade children. A possible interpretation of these results is that reading of bi-morphemic words, which contain productive roots, is impeded by the competition from morphologically related words containing the same root. The identification of the target among these morphological competitors depends on identifying the morphemic pattern (Mishkal), which is mostly composed of vowels. Since most of the vowels are not represented in the un-pointed script, there is more interference from the competitors. Thus, the extraction of the root from bi-morphemic words can facilitate identification of the word when the script is transparent, providing information about the morphemic pattern, but when the script is non-transparent, it can create competition, and even impede identification in less skilled readers who have very little experience in reading un-pointed words.

While the specific way in which orthographic transparency interacts with morphological segmentation is unique to the Semitic script and morphology, the general finding is consistent with the results of a study that compared between French and English speaking children in 3rd and 4th grade (Casalis et al., 2015). They showed greater effects of morphology in a lexical decision task in French, which has a richer morphology and a more transparent letter-to-sound correspondence than in English which has a deeper orthography. Moreover, in contrast to French children, English speaking children showed a detrimental effect for the presence of a real root in the word. The authors suggest that this is due to competition between the full word and its components because the form of the root is better preserved in English than French (Casalis et al., 2015). This finding is consistent with our results showing a disadvantage for bi-morphemic compared to mono-morphemic words when presented in the un-pointed script, and support the interpretation that this is due to competition between morphologically related words.

### Developmental Changes in the Reliance on Morphological Segmentation

Consistent with our hypothesis, the results show more pervasive morphological effects in 2nd grade compared to 5th grade children. This is reflected in the finding of stronger effect of morphological complexity on reaction time in 2nd grade compared to 5th grade children. Although both age groups were sensitive to the words’ morphological structure, as evident by the effect of morphological complextiy on accuracy in both groups, only 2nd grade children showed slower reaction time for bi-morphemic compared to mono-morphemic words, with no effect in 5th grade children. This suggests that while the process of morphological segmentation was faster and automatic in 5th grade children it was more effortful and time consuming for young children, regardless of whether it facilitated (in the pointed script) or impedes (in the un-pointed script) reading accuracy.

Another evidence for the early effect of morphological decomposition on reading is the stronger correlation found in 2nd grade compared to 5th grade children between morpho-syntactic awareness (Shany et al., 2006), and high reading accuracy of bi-morphemic words on the experimental task. The inclusion of these morphological test scores as covariates in the analysis rendered the effect of group insignificant, indicating the important role these skills play in reading acquisition. The specificity of this correlation to bi-morphemic words supports our experimental manipulation and the suggestion that processing of bi-morphemic words relies on morphological segmentation. Importantly, the finding of these correlations only in 2nd grade children suggests that the extraction of roots and morphemic patterns contributes to word identification more in these young unskilled readers compared to the older children. It is consistent with the idea that morphological segmentation in 5th graders is automatic and less demanding, and hence identification of bi-morphemic words depends less on the explicit morpho-syntactic awareness.

Our results are consistent with previous studies in Italian, using the oral naming paradigm, that showed stronger morphological effects in young or poor readers compared to adult skilled readers (Burani et al., 2008; Marcolini et al., 2011). In a similar vein, the morphemic status of French words facilitated spelling in poor but not in skilled readers (Quemart and Casalis, 2017). In Chinese, a morpho-syllabic language, morphological awareness is associated with good reading skills already in kindergarten and 2nd grade children (McBride-Chang et al., 2003). These findings show that when the morphological structure is salient and prevalent in the language, it can compensate for low phonological and decoding skills in young and poor readers.

These findings appear to contradict the claim for a developmental *increase* in the reliance on morphological segmentation skills during the elementary school years (Carlisle, 1988; Tyler and Nagy, 1989; Kieffer and Lesaux, 2012; Nagy et al., 2014; Sparks and Deacon, 2015). Some of the studies showing a developmental increase in reliance on morphological awareness explain it by an increase in the proportion of derivationally complex words children encounter as they mature and become more fluent in reading languages such as Dutch (Rispens et al., 2008) and English (Adams, 1990; Anglin, 1993; Singson et al., 2000). In contrast to this claim, derivationaly complex words are ubiquitous in the Hebrew language, and are thus very common and easily decomposed in the spoken language even in young children (Berman, 1982; Ravid and Malenky, 2001). Moreover, the morphological structure of words in the Hebrew orthography is relatively transparent because the root consonants appear as a continuous string, only interrupted occasionally by a narrow vowel letter (Ravid and Malenky, 2001; Vaknin-Nusbaum et al., 2015). These properties of the Hebrew language may explain the early development of sensitivity to the root in reading Hebrew derived words.

Indeed, our results are consistent with previous studies in Hebrew, showing that 2nd grade children already show explicit awareness of roots and patterns in *written* words (Ravid and Schiff, 2006), and this awareness is correlated with their word recognition skills (Vaknin-Nusbaum et al., 2015). Even in 1st grade Hebrew speaking children show significant contribution of morphological awareness to word reading (Schechter and Katzir, unpublished). When reading unfamiliar pseudo-words 2nd grade children rely on their morphemic structure for correct pronunciation (Shany et al., 2012). Altogether our results are consistent with the claims of the Triplex model for reading Hebrew (Share and Bar-On, 2017), suggesting that by the end of 2nd grade Hebrew speaking children rely less on low-level sub-lexical phonology, and more on higher-order lexical and morpho-orthographic knowledge. Moreover, studies in Hebrew speaking children that showed a continued increase in morphological segmentation from grade 2 to higher school grades used unfamiliar words and pseudowords, rather than real words (Bar-On and Ravid, 2011), or examined the role of morpho-syntactic information when reading sentences (Share and Bar-On, 2017). The current study shows that when reading real familiar single words performance of 5^th^ grade children is less dependent on their morphological segmentation skills compared to 2nd grade Hebrew speaking children.

### Limitations

Our study focused on reading of single words in order to control for word characteristics while testing the interaction with orthographic transparency. Nevertheless, the use of sentences which require more reliance on morpho-syntactic cues may show a continued developmental increase in the reliance on morphological segmentation in 5th grade children, and could even reverse the effect of orthographic transparency. Moreover, our study used nouns in order to manipulate the number of morphemes in the word, because the Hebrew nominal system includes both bi-morphemic (derivational) and mono-morphemic words, whereas verbs are all bi-morphemic, and their morphology is more systematic. This raises the possibility that decomposition of verbs may be more beneficial for reading across all groups and conditions.

Finally, our analyses showed no correlations between reading in the experimental task and phonological awareness, as measured by the phoneme-omission task in the Alef-Taf battery (Shany et al., 2006), in any of the groups. This stands in contrast to the ubiquitous evidence for the role of phonological awareness in reading in both Hebrew and other languages (Goswami, 1991; Bentin and Leshem, 1993; Ziegler et al., 2010). One possibility is the high level of performance on the phoneme omission test of even 2nd grade children in our sample. The average no. of errors in our sample was around 1 standard deviation lower than the average in the norms, in both groups (see **Table 2**).

## Conclusion

The current study shows that children as young as 2nd grade engage in morphological segmentation of Hebrew words composed of a root and a morphemic pattern. This process is effortful and time consuming for 2nd graders and automatic for 5th grade children. Yet, for all children this morphological structure facilitates word identification when the script is transparent. When the script is not transparent, and the vowels that constitute the morphemic pattern are not fully represented, the presence of a root impedes word recognition presumably due to competition from morphologically related words that have the same root and a different morphemic pattern. These results stand in contrast to the idea that morphological segmentation of derived words is applied to compensate for the under-specification of vowels in a non-transparent orthography (Frost, 2006; Bar-On and Ravid, 2011; Vaknin-Nusbaum and Miller, 2011). The current study, which was the first to test this idea empirically in a within-subject experiment, showed an opposite effect of orthographic transparency on morphological segmentation, with greater benefit from morphological segmentation in the transparent orthography. These results are consistent with previous studies showing reliance on morphological segmentation even in transparent orthographies such as Italian and French (Burani et al., 2008; Casalis et al., 2015). Together these findings show that morphological segmentation and decoding of single letters do not constitute alternative-competitive pathways to word recognition. They are consistent with the Grain-Size Hypothesis, suggesting that orthographic units of multiple sizes are mapped simultaneously to their phonological and meaning representations, and together they converge during word identification (Ziegler and Goswami, 2005).

Our results further show that 2nd grade Hebrew speaking children engage in morphological segmentation of derived words and this affects their reading more than for 5th grade children. We suggest that this early peak in the reliance on morphological segmentation is related to the structure of the Hebrew language and orthography that facilitates such decomposition.

## Ethics Statement

This study was carried out in accordance with the recommendations of the Ethics committee of the University of Haifa, and the Ethics committee of the Ministry of Education in Israel, with written informed consent from all subjects. All subjects gave written informed consent in accordance with the Declaration of Helsinki.

## Author Contributions

LH was involved in running the experiment, analyzing data and writing the manuscript. YW was involved in designing the experiment, developing the stimuli, overseeing participant recruitment and administration of the study and writing the manuscript. TK was involved in designing the experiment and writing the manuscript. TB was involved in designing the experiment, developing the stimuli, supervision of LH and YW, analyzing the data and writing the manuscript.

## Conflict of Interest Statement

The authors declare that the research was conducted in the absence of any commercial or financial relationships that could be construed as a potential conflict of interest.

## References

[B1] Adams M. J. (1990). *Beginning to Read.* Cambrdige, MA: MIT Press.

[B2] Anglin J. M. (1993). Vocabulary development- a morphological analysis. *Monogr. Soc. Res. Child Dev.* 58 1–166. 10.2307/1166112

[B3] Bar-On A. (2010). *The Role of Linguistic Knowledge in Learning to Read Non-voweled Hebrew.* Tel Aviv: Tel Aviv University.

[B4] Bar-On A. Ravid D. (2011). Morphological analysis in learning to read pseudowords in Hebrew. *Appl. Psycholinguist.* 32 553–581. 10.1017/S014271641100021X

[B5] Ben-Dror I. Frost R. Bentint S. (1995). Orthographic representation and phonemic segmentation in skilled readers - a cross-language comparison. *Psychol. Sci.* 6 176–181. 10.1111/j.1467-9280.1995.tb00328.x

[B6] Bentin S. Feldman L. B. (1990). The contribution of morphological and semantic relatedness to repetition priming at short and long lags - evidence from Hebrew. *Q. J. Exp. Psychol. A* 42 693–711. 10.1080/14640749008401245 2287760

[B7] Bentin S. Frost R. (1987). Processing lexical ambiguity and visual word recognition in a deep orthography. *Mem. Cogn.* 15 13–23. 10.3758/BF03197708 3821487

[B8] Bentin S. Leshem H. (1993). On the interaction between phonological awareness and reading acquisition - its a 2-way street. *Ann. Dyslexia* 43 125–148. 10.1007/BF02928178 24233989

[B9] Berman R. A. (1982). Verb-pattern alternation - the interface of morphology, syntax and semantics in Hebrew child language. *J. Child Lang.* 9 169–191. 10.1017/S030500090000369X 7061628

[B10] Bertram R. Laine M. Karvinen K. (1999). The interplay of word formation type, affixal homonymy, and productivity in lexical processing: evidence from a morphologically rich language. *J. Psycholinguist. Res.* 28 213–226. 10.1023/A:1023200313787

[B11] Bowers P. N. Kirby J. R. Deacon H. (2010). The effects of morphological instruction on literacy skills: a systematic review of the literature. *Rev. Educ. Res.* 80 144–179. 10.3102/0034654309359353

[B12] Burani C. Marcolini S. De Luca M. Zoccolotti P. (2008). Morpheme-based reading aloud: evidence from dyslexic and skilled Italian readers. *Cognition* 108 243–262. 10.1016/j.cognition.2007.12.010 18262178

[B13] Carlisle J. F. (1988). Knowledge of derivational morphology and spelling ability in 4th, 6th, and 8th graders. *Appl. Psycholinguist.* 9 247–266. 10.1017/S0142716400007839

[B14] Carlisle J. F. (2000). Awareness of the structure and meaning of morphologically complex words: impact on reading. *Read. Writ.* 12 169–190. 10.1023/A:1008131926604

[B15] Carlisle J. F. (2010). Effects of instruction in morphological awareness on literacy achievement: an integrative review. *Read. Res. Q.* 45 464–487. 10.1598/RRQ.45.4.5

[B16] Carlisle J. F. Fleming J. (2003). Lexical processing of morphologically complex words in the elementary years. *Sci. Stud. Read.* 7 239–253. 10.1207/S1532799XSSR0703_3

[B17] Carlisle J. F. Nomanbhoy D. M. (1993). Phonological and morphological awareness in 1st-graders. *Appl. Psycholinguist.* 14 177–195. 10.1017/S0142716400009541

[B18] Carlisle J. F. Stone C. A. (2005). Exploring the role of morphemes in word reading. *Read. Res. Q.* 40 428–449. 10.1598/RRQ.40.4.3

[B19] Casalis S. Louis-Alexandre M. F. (2000). Morphological analysis, phonological analysis and learning to read French: a longitudinal study. *Read. Writ.* 12 303–335. 10.1023/A:1008177205648

[B20] Casalis S. Quemart P. Duncan L. G. (2015). How language affects children’s use of derivational morphology in visual word and pseudoword processing: evidence from a cross-language study. *Front. Psychol.* 6:452. 10.3389/fpsyg.2015.00452 25932018 PMC4399200

[B21] Clahsen H. Eisenbeiss S. Sonnenstuhl-Henning I. (1997). Morphological structure and the processing of inflected words. *Theor. Linguist.* 23 201–250. 10.1515/thli.1997.23.3.201

[B22] Clark E. V. Cohen S. R. (1984). Productivity and memory for newly formed words. *J. Child Lang.* 11 611–625. 10.1017/S0305000900005985 6501468

[B23] Cohen-Mimran R. (2009). The contribution of language skills to fluency: a comparison of two orthographies for Hebrew. *J. Child Lang.* 36 657–672. 10.1017/S0305000908009148 19134231

[B24] Deacon S. H. Kirby J. R. (2004). Morphological awareness: just “more phonological”? The roles of morphological and phonological awareness in reading development. *Appl. Psycholinguist.* 25 223–238. 10.1017/S0142716404001110

[B25] Duncan L. G. Casalis S. Colé P. (2009). Early metalinguistic awareness of derivational morphology: observations from a comparison of English and French. *Appl. Psycholinguist.* 30 405–440. 10.1017/S0142716409090213

[B26] Fowler A. E. Liberman I. Y. (1995). “The role of phonology and orthography in morphological awareness,” in *Morphological Aspects of Language Processing*, ed. Feldman L. B. (Hillsdale, NJ: Erlbaum), 157–188.

[B27] Frost R. (2006). Becoming literate in Hebrew: the grain size hypothesis and Semitic orthographic systems. *Dev. Sci.* 9 439–440. 10.1111/j.1467-7687.2006.00523.x 16911440

[B28] Frost R. (2012). Towards a universal model of reading. *Behav. Brain Sci.* 35 263–329. 10.1017/S0140525X11001841 22929057 PMC3677812

[B29] Frost R. Forster K. Deutsch A. (1997). What can we learn from the morphology of Hebrew? A masked-priming investigation of morphological representation. *J. Exp. Psychol. Learn. Mem. Cogn.* 23 829–856. 10.1037/0278-7393.23.4.829 9265076

[B30] Gillis S. Ravid D. (2006). Typological effects on spelling development: acrosslinguistic study of Hebrew and Dutch. *J. Child Lang.* 33 621–659. 10.1017/S0305000906007434 17017281

[B31] Goswami U. (1991). Learning about spelling sequences - the role of onsets and rimes in analogies in reading. *Child Dev.* 62 1110–1123. 10.2307/1131156

[B32] Harel-Koren D. (2007). *The Effectiveness of Orthographic Representations of Vowels Signs in Hebrew: Developmental Aspect.* Master thesis, Department of Learning Disabilities, University of Haifa, Haifa..

[B33] Kieffer M. J. Lesaux N. K. (2012). Direct and indirect roles of morphological awareness in the English reading comprehension of native English, Spanish, Filipino, and Vietnamese speakers. *Lang. Learn.* 62 1170–1204. 10.1111/j.1467-9922.2012.00722.x

[B34] Koriat A. (1984). “Reading without vowels: lexical access in Hebrew,” in *Attention and Performance X: Control of Language Processes*, eds Bouma H. Bouwhuis D. G. (Hillsdale, NJ: Lawrence Erlbaum Associates), 227–242.

[B35] Koriat A. (1985). Lexical access for low- and high-frequency words in Hebrew. *Mem. Cogn.* 13 37–44. 10.3758/BF03198441 4010512

[B36] Kuo L. J. Anderson R. C. (2006). Morphological awareness and learning to read: a cross-language perspective. *Educ. Psychol.* 41 161–180. 10.1207/s15326985ep4103_3

[B37] Mahony D. Singson M. Mann V. (2000). Reading ability and sensitivity to morphological relations. *Read. Writ.* 12 191–218. 10.1023/A:1008136012492

[B38] Marcolini S. Traficante D. Zoccolotti P. Burani C. (2011). Word frequency modulates morpheme-based reading in poor and skilled Italian readers. *Appl. Psycholinguist.* 32 513–532. 10.1017/S0142716411000191

[B39] Marslen-Wilson W. Komisarjevsky Tyler L. Waksler R. Older L. (1994). Morphology and meaning in the English mental lexicon. *Psychol. Rev.* 101 3–33. 10.1037/0033-295X.101.1.3

[B40] McBride-Chang C. Shu H. Zhou A. Wagner R. K. (2003). Morphological awareness uniquely predicts young children’s Chinese character recognition. *J. Educ. Psychol.* 95 743–751. 10.1037/0022-0663.95.4.743

[B41] Melby-Lervag M. Lyster S. A. Hulme C. (2012). Phonological skills and their role in learning to read: a meta-analytic review. *Psychol. Bull.* 138 322–352. 10.1037/a0026744 22250824

[B42] Merkx M. Rastle K. Davis M. H. (2011). The acquisition of morphological knowledge investigated through artificial language learning. *Q. J. Exp. Psychol.* 64 1200–1220. 10.1080/17470218.2010.538211 21271463

[B43] Nagy W. Berninger V. Abbott R. Vermeulen K. (2003). Relationship of morphology and other language skills to literacy skills in at-risk second-grade readers and at-risk fourth-grade writers. *J. Educ. Psychol.* 95 730–742. 10.1037/0022-0663.95.4.730

[B44] Nagy W. E. Carlisle J. F. Goodwin A. P. (2014). Morphological knowledge and literacy acquisition introduction. *J. Learn. Disabil.* 47 3–12. 10.1177/0022219413509967 24219917

[B45] Navon D. Shimron J. (1981). Does word naming involve grapheme-to-phoneme translation? Evidence from Hebrew. *J. Verbal Learn. Verbal Behav.* 20 97–109. 10.1016/S0022-5371(81)90334-0

[B46] Nunes T. Bryant P. Olsson J. (2003). Learning morphological and phonological spelling rules: an intervention study. *Sci. Stud. Read.* 7 289–307. 10.1207/S1532799XSSR0703_6

[B47] Quemart P. Casalis S. (2017). Morphology and spelling in French students with dyslexia: the case of silent final letters. *Ann. Dyslexia* 67 85–98. 10.1007/s11881-016-0133-3 27553683

[B48] Quemart P. Casalis S. Colé P. (2011). The role of form and meaning in the processing of written morphology: a priming study in French developing readers. *J. Exp. Child Psychol.* 109 478–496. 10.1016/j.jecp.2011.02.008 21419425

[B49] Rastle K. Davis M. H. (2008). Morphological decomposition based on the analysis of orthography. *Lang. Cogn. Process.* 23 942–971. 10.1080/01690960802069730

[B50] Ravid D. (1996). Accessing the mental lexicon: evidence from incompatibility between representation of spoken and written morphology. *Linguistics* 34 1219–1246. 10.1515/ling.1996.34.6.1219

[B51] Ravid D. Malenky A. (2001). Awareness of linear and nonlinear morphology in Hebrew: a developmental study. *First Lang.* 21 25–56. 10.1177/014272370102106102

[B52] Ravid D. Saban R. (2008). “Syntactic and meta-syntactic skills in the school years: a developmental study in Hebrew,” in *Language Education in Israel: Papers in Honor of Elite Olshtain*, eds Kupferberg I. Stavans A. (Jerusalem: Magnes Press), 75–110.

[B53] Ravid D. Schiff R. (2006). Roots and patterns in Hebrew language development: evidence from written morphological analogies. *Read. Writ.* 19 789–818. 10.1007/s11145-006-9004-3

[B54] Rispens J. E. McBride-Chang C. Reitsma P. Rispens J. McBride-Chang C. Reitsma P. (2008). Morphological awareness and early and advanced word recognition and spelling in Dutch. *Read. Writ.* 21 587–607. 10.1007/s11145-007-9077-7

[B55] Schiff R. Ravid D. (2004). Vowel representation in written Hebrew: phonological, orthographic and morphological contexts. *Read. Writ.* 17 241–265. 10.1023/B:READ.0000017668.48386.90

[B56] Shankweiler D. Crain S. Katz L. Fowler A. E. Liberman A. M. Brady S. A. (1995). Cognitive profiles of reading-disabled children: comparison of language skills in phonology, morphology, and syntax. *Psychol. Sci.* 6 149–156. 10.1111/j.1467-9280.1995.tb00324.x

[B57] Shany M. Bar-On A. Katzir T. (2012). Reading different orthographic structures in the shallow-pointed Hebrew script: a cross-grade study in elementary school. *Read. Writ.* 25 1217–1238. 10.1007/s11145-011-9314-y

[B58] Shany M. Lachman D. Shalem Z. Bahat A. Zeiger T. (2006). *Alef-Taf. Evaluation System for the Diagnosis of Disability in the Reading and Writing Process According to National Norms.* Holon: Yesod Publishing.

[B59] Shany M. Share D. L. (2011). Subtypes of reading disability in a shallow orthography: a double dissociation between accuracy-disabled and rate-disabled readers of Hebrew. *Ann. Dyslexia* 61 64–84. 10.1007/s11881-010-0047-4 21108026

[B60] Share D. L. (1995). Phonological recoding and self-teaching: sine qua non of reading acquisition. *Cognition* 55 151–218; discussion 219–226. 10.1016/0010-0277(94)00645-2 7789090

[B61] Share D. L. Bar-On A. (2017). Learning to read a Semitic Abjad: the triplex model of Hebrew reading development. *J. Learn. Disabil.* 10.1177/0022219417718198 [Epub ahead of print]. 28703637

[B62] Shimron J. Navon D. (1982). The dependence on graphemes and on their translation to phonemes in reading: a developmental perspective. *Read. Res. Q.* 17 210–228. 10.2307/747484

[B63] Shimron J. Sivan T. (1994). Reading proficiency and orthography evidence from Hebrew and English. *Lang. Learn.* 44 5–27. 10.1111/j.1467-1770.1994.tb01447.x

[B64] Singson M. Mahony D. Mann V. (2000). The relation between reading ability and morphological skills: evidence from derivational suffixes. *Read. Writ.* 12 219–252. 10.1023/A:1008196330239

[B65] Sparks E. Deacon S. H. (2015). Morphological awareness and vocabulary acquisition: a longitudinal examination of their relationship in English-speaking children. *Appl. Psycholinguist.* 36 299–321. 10.1017/S0142716413000246

[B66] Tolchinsky L. Levin I. Aram D. McBride-Chang C. (2012). Building literacy in alphabetic, abjad and morphosyllabic systems. *Read. Writ.* 25 1573–1598. 10.1007/s11145-011-9334-7

[B67] Treiman R. Cassar M. (1996). Effects of morphology on children’s spelling of final consonant clusters. *J. Exp. Child Psychol.* 63 141–170. 10.1006/jecp.1996.0045 8812040

[B68] Tyler A. Nagy W. (1989). The acquisition of English derivational morphology. *J. Mem. Lang.* 28 649–667. 10.1016/0749-596X(89)90002-8

[B69] Vaknin-Nusbaum V. Miller P. (2011). The importance of vowel diacritics for the temporary retention of high and low frequency Hebrew words of varying syllabic length. *Mem. Cogn.* 39 516–526. 10.3758/s13421-010-0026-3 21264614

[B70] Vaknin-Nusbaum V. Sarid M. Raveh M. Nevo E. (2015). Morphonological awareness. *Read. Writ.* 29 229–244. 10.1007/s11145-015-9587-7

[B71] Vaknin-Nusbaum V. Sarid M. Raveh M. Nevo E. (2016a). The contribution of morphological awareness to reading comprehension in early stages of reading. *Read. Writ.* 29 1915–1934. 10.1007/s11145-016-9658-4

[B72] Vaknin-Nusbaum V. Sarid M. Shimron J. (2016b). Morphological awareness and reading in second and fifth grade: evidence from Hebrew. *Read. Writ.* 29 229–244. 10.1007/s11145-015-9587-7

[B73] Vellutino F. R. Scanlon D. M. (1987). Phonological coding, phonological awareness, and reading-ability - evidence from a longitudinal and experimental-study. *Merrill Palmer Q.* 33 321–363.

[B74] Wechsler D. (1994). *Wechsler Intelligence Scale for Children (WISC-III)*, 3rd Edn Cleveland, OH: Psychological Corporation.

[B75] Weiss Y. Katzir T. Bitan T. (2015a). Many ways to read your vowels - neural processing of diacritics and vowel letters in Hebrew. *Neuroimage* 121 10–19. 10.1016/j.neuroimage.2015.07.029 26188258

[B76] Weiss Y. Katzir T. Tali B. (2015b). The effects of orthographic transparency and familiarity on reading Hebrew words in adults with and without dyslexia. *Ann. Dyslexia* 65 84–102. 10.1007/s11881-015-0100-4 25911275

[B77] Ziegler J. C. Bertrand D. Tóth D. Csépe V. Reis A. Faísca L. (2010). Orthographic depth and its impact on universal predictors of reading: a cross-language investigation. *Psychol. Sci.* 21 551–559. 10.1177/0956797610363406 20424101

[B78] Ziegler J. C. Goswami U. (2005). Reading acquisition, developmental dyslexia, and skilled reading across languages: a psycholinguistic grain size theory. *Psychol. Bull.* 131 3–29. 10.1037/0033-2909.131.1.3 15631549

[B79] Ziegler J. C. Stone G. O. Jacobs A. M. (1997). What’s the pronounciation for _OUGH and the spelling for vertical bar u vertical bar? A database for computing feedforward and feedback consistency in English. *Behav. Res. Methods Instrum. Comput.* 29 600–618. 10.3758/BF03210615

