# Supplementary material for: Orthographic Transparency Enhances Morphological Segmentation in Children Reading Hebrew Words
Source: Front Psychol. 2018 Jan 19;8:2369. doi: 10.3389/fpsyg.2017.02369 (PMC5780401; doi:10.3389/fpsyg.2017.02369)
Supplement: Supplementary file 1 [file Table_1.docx]

**Supplementary material**

|  | **2^nd^ grade (n=28)**  Mean S.D. | **5^th^ grade (n=29)**  Mean S.D. |
| --- | --- | --- |
| **Reading Pseudo-words**  (% errors) | **13.85 (13.66)**  38.57 (24.51) | **24.76 (20.85)**  There are no norms |
| **Reading Pseudo-words** (number per minute) | **24.86 (5.00)**  17.87 (8.89) | **33.04 (5.83)**  23.08 (10.36) |
| **Reading words**  (% errors) | **6.29 (6.13)**  22.65 (18.41) | **5.90 (4.32)**  12.08 (10.93) |
| **Reading words**  (number per minute) | **45.20 (10.36)**  30.12 (15.41) | **59.19 (14.30)**  55.74 (19.93) |

***Table S1.*** *Participants’ average raw score in the screening standardized tests.* The mean (and standard deviation in parenthesis) of the sample appear in bold in the upper row and the scores of the norms sample of the Alef-Taf appear in regular type in the lower row. The scores are based on different age-appropriate lists of items, so the raw scores are not comparable across age groups.
